# Use of a Rotating Square Spatial-Frequency Filter to Map the Optical Path Length Variation in Microscopic Biological Samples

**DOI:** 10.3390/s22051842

**Published:** 2022-02-25

**Authors:** Ignacio Iglesias

**Affiliations:** Departamento de Física, CEIR Campus Mare Nostrum (CMN), Universidad de Murcia, 30100 Murcia, Spain; ignacio.iglesias@um.es; Tel.: +34-868-887-220

**Keywords:** microscopy, quantitative microscopy, wavefront sensing

## Abstract

Gradient images can be obtained using a rotating square mask to filter the angular spectra of the wavefront generated by a complex transmittance object. This method can be applied to measure the three-dimensional structure of microscopic biological samples through the relationship of the phase with the optical path length. This work describes the implementation of a system using an inverted optical microscope and shows the experimental results of phase maps generated by boar sperm cells.

## 1. Introduction

Phase-contrast microscopes allow for the observation of intensity variations that are related, in a complex way, to the wavefronts generated by transparent samples that otherwise appear flat under a simple microscope. A wavefront’s shape depends on the optical path length (OPL) and is the product of the path length followed by the light and the refractive index. The determination of the OPL or quantitative phase microscopy is more complicated and has only been possible since the advent of electronic imaging. Over the years, several techniques have been proposed using digital image registration and processing [1]. Some are based on the acquisition of interference patterns generated by the sample and a reference beam. Other methods—inspired by the wavefront sensing techniques developed in astronomy for the determination and compensation of atmosphere-induced wave aberrations—do not require a reference beam and thus have different advantages over the interferometric means. These techniques do not access the wavefront directly but rather at the first [2,3] or second [4,5] derivative.

Among the systems that measure the first derivative, the pyramid sensor [6] has interesting characteristics, such as high lateral resolution and sensitivity. This had made it the sensor of choice in state-of-the-art, high-performance telescopes. In microscopy, the same properties are also convenient both for aberration correction in high-resolution microscopy [7,8] and quantitative phase microscopy [9].

The pyramid sensor derives its name from the use of a glass pyramid. The absence of rotational symmetry makes it difficult to fabricate this component with enough precision. In addition, if only one image sensor is used, it must accommodate four images, each containing the full lateral extension of the wavefront with the consequent reduction in the sampling.

As an alternative, we have recently shown [10] that the pyramid microscopy technique can be implemented using a rotating mask placed on a Fourier transform plane of the microscope’s camera port image instead of a glass pyramid. An opaque mask is much easier to fabricate and allows the use of the complete image sensor area, which increases the sensor lateral resolution. In the same cited work, we presented a procedure to correct the sensor characteristic nonlinear gain using calibrated polystyrene microspheres and obtaining a response matrix that incorporates the effect of the structure of the illumination source. An additional advantage of the rotating mask approach is that it allows for the acquisition of an ensemble of distinct images for different incremental rotations, improving the SNR by averaging.

In the present work, we extend our previous results by showing the practical application of the Fourier plane rotating mask method in quantitative phase microscopy of biological samples with sub-cellular resolution. In addition, we incorporated a nanopositioner into the system to show the impact of the sample plane position on the phase measurements.

For insight into how this method works, consider a plane wave that traversed a phase object. The wavefront generated is decomposed into local plane wavelets with different orientations in space, each one represented by a wave vector along the normal. A positive lens will rotate the wave vectors, so if the wavefront is a perfect plane, all will be directed to the optical axis at the back focal plane (the Fourier plane). If the wavefront has a local slope, the vector associated with this region will cross the back focal plane at specific coordinates different from the optical axis position. In this way, determining the coordinates at the Fourier plane is equivalent to determining the local slope. The quadrant-sensor detection strategy is used to measure the coordinates, i.e., the Fourier plane is divided into quadrants, and the intensity of each is compared to determine the Cartesian coordinates. The intensity detection is not in the focal plane but in a plane conjugated with the input wavefront by a second lens. The measurement is sequential, meaning one image is captured for each quadrant. In this way, the intensity at a given point of the conjugate plane represents the corresponding quadrant intensity of the local wave vector at this point. However, the coordinates are not entirely determined in this way, only the sign. To measure the magnitude, the energy associated with a vector corresponding to a local wavelet must be spread in the four quadrants to calculate the value of the intensity distribution in each quadrant. Instead of using an illuminated plane wave for this (a wave that can be seen as emerging from a point source at infinite), an extended symmetrical source at infinite is used.

The use of high numerical aperture (NA) objectives is a requirement for detailed imaging of cells. However, the small depth of field that characterizes these objectives makes that, even for small axial sample displacements, defocus significantly alters the phase maps. As happens in digital holographic microscopy, the correct focus plane is not obvious from a simple camera image before processing [11]. To manage this issue for posterior comparison, a nanopositioner is installed to hold the sample for registering phase maps with small variations in the distance between the sample and the microscope objective.

## 2. Materials and Methods

**Figure 1:**
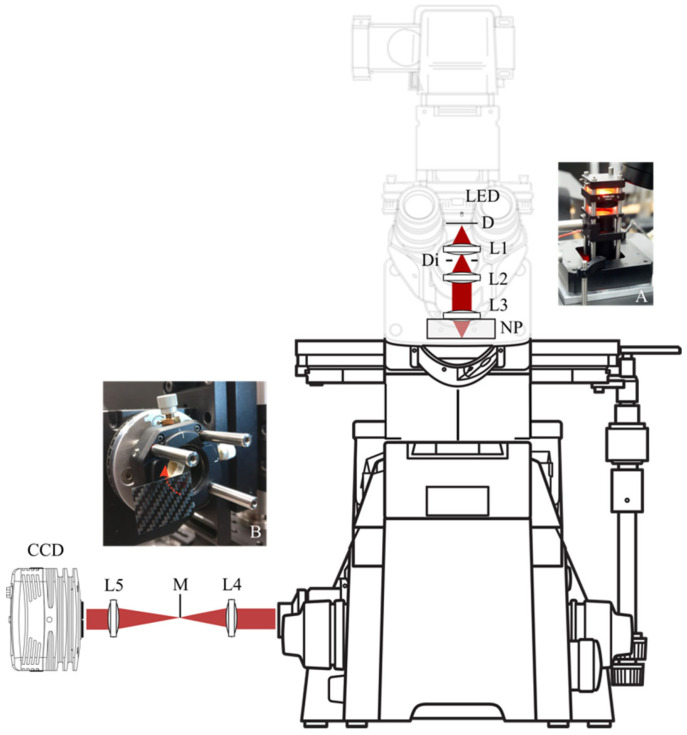
Implementation of the rotating mask phase imaging using a commercial microscope (Nikon TE2000). LED is a red LED; D is a diffuser; Di is a diaphragm; L1–L5 are lenses; NP is a three-axis nanopositioner stage; M is a carbon-fiber mask; CCD is a digital image sensor. Inset (**A**) shows the alternative illumination, and Inset (**B**) shows the mask at the XY translation stage.

Figure 1 shows a scheme of the Nikon Eclipse TE2000 microscope used as a platform for the system. The microscope mounts a Nikon, CFI Plan Apochromat 100× oil immersion objective. The Köhler built-in halogen lamp illumination is not homogeneous enough for this purpose and is bypassed by an alternative system to regularize the intensity distribution (Figure 1A). A red LED with a 625 nm central wavelength is installed before a diffuser (D) and a lens (L1) of 30 mm focal length (Thorlabs (Newton, NJ, USA), AC254-030-A-ML) is used to produce an image of the diffuser at infinity. A diaphragm (Di) controls the illumination extension, and a 4f system (Thorlabs, MAP103030-A) that is represented as lenses L2 and L3 with 30 mm focal lengths images the exit pupil of lens L1 on the sample plane. The microscope stage is equipped with a three-axis piezo nanopositioner (NP) (Physik Instrumente (Karlsruhe, Germany), P-733.3DD) used to precisely control the sample axial position. After the microscope camera port, two lenses, L4 with a 75 mm focal length (Thorlabs, AC254-075-A-ML) and L5 with a 150 mm focal length (Thorlabs, AC254-150-A-ML), relay the image onto a CCD sensor (Thorlabs, 1500M-CL-TE). The first lens, L4, is placed at a distance from the microscope camera port image plane equal to its front focal length, forming the Fourier transform at the back focal plane. In this plane, a mask (M) is placed (Figure 1B), filtering the angular spectrum of the microscope’s camera port image. Instead of using a brittle gold-plated coverslip [10] with edges that are not entirely straight and cannot be polished without damaging the gold layer, the mask here is made from a 0.3 mm thick carbon fiber plate that was cut manually into a square before having its edges wet-sanded. For the rotation of the mask, a rotation stage (Physik Instrumente, DT-80) is used (Figure 1B).

**Figure 2:**
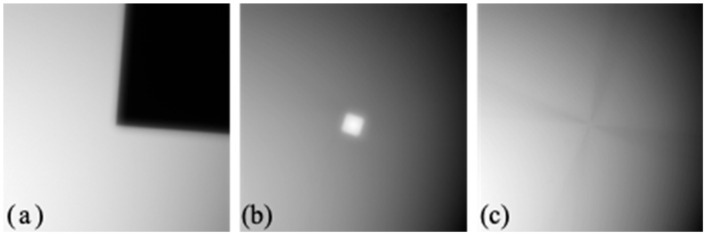
Images (901 px × 901 px) involved in the mask alignment procedure: (**a**) Image of the mask obtained with an auxiliary lens (the angle of the mask corresponds to the offset angle); (**b**) average of several images obtained rotating the mask when the corner was mislaid with respect to the rotation axis and (**c**) when it was in the correct position.

It is critical to adjust the mask corner to coincide with the rotation axis of the rotation stage to correctly occlude quadrants when rotating. For this purpose, the mask is glued on a manual XY lateral translation stage (Thorlabs, CXY1) and assembled to the rotation stage, as seen in Inset B of Figure 1. To facilitate alignment, a supplementary lens of 125 mm focal length (Thorlabs, AC254-125-A-ML) and an external diffuse illumination source (not shown in Figure 1) are temporarily placed between the lens L5 and the Fourier plane to image the mask on the CCD. Figure 2a shows an image of the mask obtained in this way.

An average of several images is registered by rotating the mask to find the rotation axis that must coincide with the mask corner. As shown in Figure 2b, a bright area (or dark area, depending on the initial position) appears when the corner is not centered; then, the corner of the mask is relocated to the center of this area. The procedure is repeated until the best position is reached (Figure 2c). The accuracy of this procedure is limited by the precision of the manual translating stage and the resolution of the mask image.

**Figure 3:**
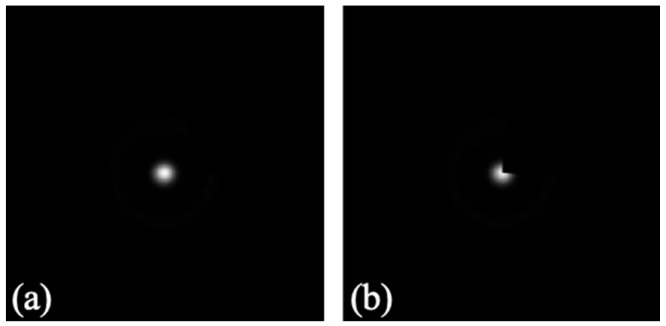
(**a**) Illumination source intensity distribution on the Fourier plane captured with the auxiliary lens; (**b**) illumination source intensity distribution on the Fourier plane captured with the auxiliary lens with the mask in place.

After removing the external light source, the auxiliary lens is also used for evaluating the structure of the illumination and for adjusting the diaphragm (Di). Figure 3a shows the source intensity distribution on the Fourier plane without the mask and, in Figure 3b, with the mask at its final position. For the best sensor response, the source must be extended to its fullest extent possible [9]. For this system, the diaphragm is slightly closed to keep spurious light from entering the system as it does when fully opened, which would reduce the illumination’s homogeneity.

The method does not require rotating the mask for alignment with the CCD sensor but only to determine its offset angle. The angle measurement tool of the image distribution of Fiji [12] is used on the image in Figure 2a to obtain the initial value (5° in this case).

The wavefront gradient signal, $\left[S_{x},S_{y}\right]$, is obtained from the acquired images using the expression [10]

(1) $$\left[\begin{array}{c} S_{x}\\ S_{y} \end{array}\right]=\frac{1}{I_{T}}\left[\begin{array}{cc} \cos\theta & -\sin\theta \\ \sin\theta & \cos\theta \end{array}\right]\left[\begin{array}{c} I\left(\theta +270^{\circ }\right)+I\left(\theta +180^{\circ }\right)-I\left(\theta +90^{\circ }\right)-I\left(\theta \right)\\ I\left(\theta +270^{\circ }\right)-I\left(\theta +180^{\circ }\right)-I\left(\theta +90^{\circ }\right)+I\left(\theta \right) \end{array}\right]$$

where I is an image acquired for a given mask angle, $I_{T}=I\left(\theta \right)+I\left(\theta +90^{\circ }\right)+I\left(\theta +180^{\circ }\right)+I\left(\theta +270^{\circ }\right)$, and θ is the mask offset angle, respecting the image sensor that provides the x and y reference axes. Equation (1) shows that four images are required, which correspond to increments of 0°, 90°, 180°, and 270° for the offset angle. However, the free rotation of the mask allows for a more numerous ensemble to be acquired by changing the offset angle. This is useful for increasing the number of measures of a single wavefront reducing the noise produced by averaging. In practice, the mask rotates in steps (we arbitrarily chose 1°, although the rotation stage permits much smaller values), and groups of four images are selected among the stored images for the different offsets in the processing step.

**Figure 4:**
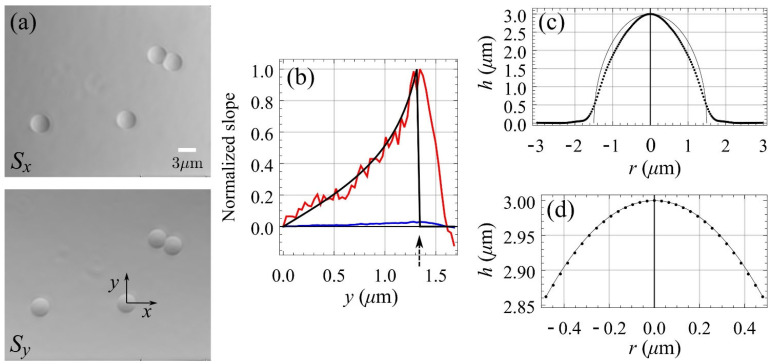
In panel (**a**), images (1156 × 961 px) of the sensor response to the wavefront gradient in the horizontal and vertical directions for polystyrene microspheres of 3 µm diameter immersed in index matching oil. Panel (**b**) presents the response along the y coordinate of panel in blue, the corrected response in red and the expected response in black, while the arrow marks the limit of the sensor response. Panel (**c**) shows the radial profile of the measured wavefront (dotted line) and the ideal wavefront (solid line). Panel (**d**) shows the radial profile of the measured wavefront (dotted line) and the ideal wavefront (solid line) for a circular region with no saturated gradient values.

Figure 4 shows the images of the raw sensor response to the wavefront gradient generated by 3 µm diameter polystyrene microspheres immersed in index matching oil in the horizontal (a) and vertical (b) directions corresponding to the average of $S_{x}$ and $S_{y}$ in Equation (1) for the different angles θ. The images are consistent with the gradient values expected in a wavefront generated by spheres with a slightly different refraction index than the immersion medium.

Given the high numerical aperture (1.4) of the immersion objective, the depth of focus is very restricted and phase changes are apparent even for a 0.1 µm sample axial position variation. In addition, a single camera image for a given mask angle does not allow for visual determination of whether the sample is in focus on the computer screen. This is addressed by registering gradient data for different sample axial positions with 0.1 µm intervals. The images in Figure 4 correspond to the axial positions that better match the expected gradients of a wavefront generated by a sphere.

The sensor response is non-linear and not uniform for different polar angles [9,13]. A calibration step is needed to correct the response before numerical integration. One isolated microsphere of Figure 4a is used to obtain the sensor response matrix, as detailed in [10], and applied to posterior raw gradient measurements. The blue line in Figure 4b shows the raw data along the positive part of the y-axis for a microsphere; the red line shows the corrected values; and the black line shows the expected data given by the derivative of the thickness at a point with coordinates $\left(0,y\right)$, i.e., $d\left(2\sqrt{r_{sphr}^{2}-y^{2}}\right)/dy$, where $r_{sphr}$ represents the microsphere radius. For the last two plots, the data were normalized to the slope value for which the sensor reached saturation (the extreme of the measurement range). This limit is marked with an arrow in Figure 4b. The Frankot–Chellappa [14] integration method is applied to the gradient measure to obtain the wavefront. The dotted line in Figure 4c shows the radial profile of the measured wavefront with the ideal radial profile as a solid line. As shown, the microsphere generates wavefront gradients outside of the sensor measurement range close to the border with the surrounding medium. The main reasons for discrepancies between the obtained and ideal wavefronts are the inclusion of this region with saturated gradient values and the objective of the integration algorithm to provide the best fit to the given slope data. As Figure 4b shows, given that the gradient is correctly determined in the area where the sensor is not saturated, if the integration algorithm is restricted to this area, the match between the expected and measured wavefronts is almost perfect, as shown in Figure 4d. Given that the structure is known for this sample, the OPL data provided by integration can be represented in length units, as shown in Figure 4c,d, even without considering the refractive indexes.

## 3. Results

Fixed boar sperm cells diluted in Beltsville Thawing Solution (BTS) were used to probe the system with a biological sample. One drop was deposited on a glass coverslip. After a few minutes, the cells stopped floating and stabilized on the glass surface. The lack of movement caused by Brownian collisions to the cells was appreciated. The dilution was high enough to easily have a single cell in the CCD field of view. With the position of the sample holder at half of the axial movement range of the nanopositioner (10 µm) and the sample approximately focused using the microscope’s focus knob, gradient images were acquired at intervals of 0.1 µm of axial increments in both directions.

To remove unwanted diffraction noise generated by light scattering dust particles on the surfaces of the optical components as well as to discount the system’s aberrations, a background reference gradient of an area without cells was registered in the field of view by moving the microscope’s manual stage. This information was used by subtracting the wavefront gradient generated by the cells from the reference gradient before applying the integration method.

**Figure 5:**
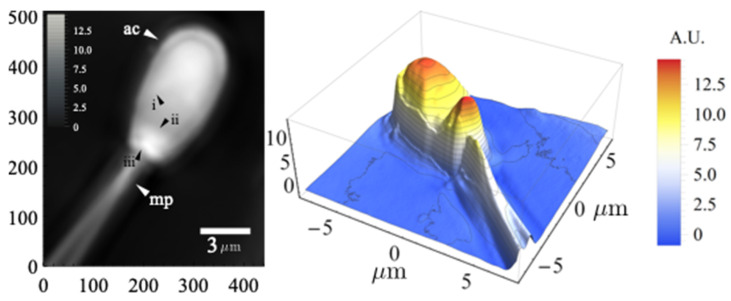
On the left panel, an image (510 × 441 px) corresponding to the optical path length in arbitrary units represented as gray tones of a boar sperm cell. The right panel shows the same information as a three-dimensional plot. The height coordinate is also in arbitrary units.

Figure 5 shows the results obtained for the axial position corresponding to the best focus. The image on the left presents the accumulated phase delay (or OPL) as a gray tone in arbitrary units. The panel on the right shows the same information as a three-dimensional plot. If the local refraction indexes of the different cell structures were known, the topography could be derived from this information.

In the data presented in Figure 5, the consequences of defocus manifested in the sperm tail after the middle piece (mp) appeared to be duplicated. This was not caused by malformation or movement during image acquisition. As shown later, it was a consequence of defocus, given that the sperm cell tail was separated from the coverslip surface in this particular sample. Several structures are visible in the sperm head. The acrosome, marked as (ac) in Figure 5, and several boundaries (black arrowheads i, ii, and iii in Figure 5) outline the extension limits of several membranes as the outer and inner acrosomal membranes and the plasma membrane. Between lines ii and iii in Figure 5, a complex structure can be seen, probably corresponding to the equatorial subsegment, a structure enriched with phosphorylated proteins that was first identified with atomic force microscopy (AFM) and studied with fluorescent markers and scanning electron microscopy (SEM) [15]. To our knowledge, the details shown here have not been obtained before with optical microscopy without using fluorescent markers.

**Figure 6:**
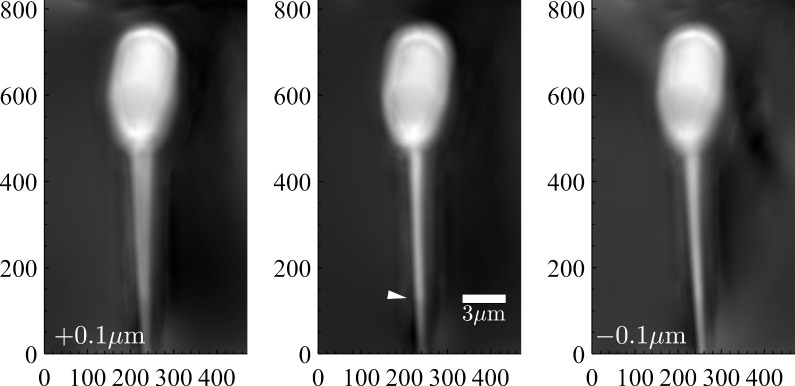
Images (821 × 471 px) presenting the optical path length as grayscale tones of a boar sperm for the different axial positions of the nanopositioner. The image at the center is for the best focus, on the left and right images obtained with ±0.1 µm axial displacements, respectively.

Figure 6 shows OPL images for three consecutive axial displacements of the nanopositioner, illustrating the effect of defocus. The image at the center corresponds to the best focus in the series, identified after processing the whole set. The images on the left and right correspond to ±0.1 µm of relative axial displacements, respectively. In this sample, unlike the previous one, the sperm tail is in contact with the coverslip. In addition to the structures that can be identified in the head, the transition between the flagellum and the axial filament (marked with an arrowhead in the central image) can be seen.

The images of Figure 6 show that the axial position of the sample is critical, causing false duplication of the sperm tail when the displacement is in the positive direction. Two conclusions can be derived: the effect of the sample position on the OPL measurement is not symmetrical, and it manifests for changes of less than 0.1 µm for the microscope objective used.

## 4. Discussion

The rotating-mask technique measures the wavefront induced by local variations in the OPL in a sample through the acquisition and processing of a series of images obtained by spatial-frequency filtering. We showed its use by obtaining quantitative phase maps generated by boar sperm cells and the potential of this technique for assessing biological samples at the microscopic scale.

The wavefront maps reveal small cell structures, indicating that the technique can complement other more sophisticated high-resolution techniques such as AFM or SEM. In particular, the combination of this technique with shape information obtained using AFM can be useful. This can be achieved by discounting the path from the OPL data to determine the local refraction index variation in small sample regions and to infer the molecular composition.

The illumination source in the system is unpolarized. Different OPL maps will be obtained for different polarizations if the sample is anisotropic, whereby this information could also be used to characterize the sample molecular structure.

We also showed that, for high-magnification quantitative phase imaging, the focus plane is critical, and it is useful to axially scan the sample to detect artifacts caused by defocus.

The results shown are preliminary, and there is room for improvement. In the current implementation, the light traverses a grating interferometer (omitted from Figure 1 for simplicity). Its internal 4f optical system transports the microscope camera port plane to one of the interferometer exit ports from where the phase measurement system is installed, contributing to system aberrations that are mainly associated with a non-perfect optical axis alignment and heavily reducing the available light. It is expected to obtain much better results with a system attached directly to the microscope camera port, as shown in Figure 1. The available light limits the temporal bandwidth, and although this is not a problem with the cells shown, it can be critical for other samples. With limited light, substrate preparation techniques can be used to fix the cells to the glass. With enough light, image sensors can be fast enough to allow for even free rotation of the mask during registration, increasing the temporal bandwidth sufficiently to avoid special substrate preparation.

Finally, the squareness of the mask is critical but not perfect enough in the current system, as can be appreciated in Figure 2a. We have devised ways to improve the cutting of the mask for the next iteration.
